# P78 Antimicrobial resistance profile of *Corynebacterium diphtheriae* isolates from a 2023–24 outbreak in Karachi, Pakistan

**DOI:** 10.1093/jacamr/dlag102.084

**Published:** 2026-06-26

**Authors:** Sobia Muhammad Asad Khan, Javaria Ashraf, Hassan Ghayas, Seema Irfan, Najia Ghanchi, Joveria Farooqi, Kausar Jabeen, Noureen Zeeshan, Muhammad Naeem, Sadaf Zaka, Egon Ozer, Rumina Hasan

**Affiliations:** Department of Pathology and Laboratory Medicine, Aga Khan University, Karachi, Pakistan; Department of Pathology and Laboratory Medicine, Aga Khan University, Karachi, Pakistan; Department of Pathology and Laboratory Medicine, Aga Khan University, Karachi, Pakistan; Department of Pathology and Laboratory Medicine, Aga Khan University, Karachi, Pakistan; Department of Pathology and Laboratory Medicine, Aga Khan University, Karachi, Pakistan; Department of Pathology and Laboratory Medicine, Aga Khan University, Karachi, Pakistan; Department of Pathology and Laboratory Medicine, Aga Khan University, Karachi, Pakistan; Department of Pathology and Laboratory Medicine, Aga Khan University, Karachi, Pakistan; Department of Pathology and Laboratory Medicine, Aga Khan University, Karachi, Pakistan; Department of Pathology and Laboratory Medicine, Aga Khan University, Karachi, Pakistan; Northwestern University Feinberg School of Medicine, Chicago, IL, USA; Department of Pathology and Laboratory Medicine, Aga Khan University, Karachi, Pakistan; Dept of Infection Biology, London School of Hygiene and Tropical Medicine, UK

## Abstract

**Background:**

Diphtheria remains endemic in Pakistan with a notable upsurge of cases following the COVID-19 pandemic, despite on-going vaccination programmes. Antimicrobial therapy plays a key role in eradicating nasal carriage of toxigenic *Corynebacterium diphtheriae* strains in affected individuals, contributing to optimal control of the disease. Amid rising case numbers, data on antimicrobial resistance in *C. diphtheriae* from this region is limited.

**Objectives:**

To evaluate phenotypic antimicrobial susceptibility profiles and associated genomic mechanisms in *C. diphtheriae* isolates from patients with respiratory diphtheria during the 2023-2024 outbreak in Karachi.

**Methods:**

*C. diphtheriae* strains isolated between August 2023- October 2024 at a tertiary care hospital laboratory in Karachi, Pakistan were included. Demographic and clinical data were recorded (when available). Antimicrobial susceptibility testing of phenotypically confirmed *C. diphtheriae* was performed as per CLSI and EUCAST guidelines. Whole-genome sequencing was done using Illumina MiniSeq platform and diphthOscan and AMRfinderPlus tools were used to determine presence of antimicrobial resistance genes (ARGs). Cohen’s kappa test was applied to assess concordance between the phenotypic and genotypic results.

**Results:**

A total of 48 pharyngeal *C. diphtheriae* isolates were analysed. Median age of patients was 7 years and male to female ratio was 1.6:1. Phenotypically, 64.5% (*n*=31) were resistant to erythromycin and 33.3% (*n*=16) were resistant to penicillin. Alarmingly, resistance to trimethoprim/sulfamethoxazole was 100% (*n*=48). In contrast, all isolates were susceptible to meropenem. Overall, 33.3% (*n*=16) were resistant to both erythromycin and penicillin, and 48% (*n*=23) were multi-drug resistant strains. The most prevalent resistance genes were *sul1* (100%), *ermX* (72.9%) and *pbp2m* (50%). Discordance between phenotypic and genotypic resistance was observed as follows: 52% (*n*=25) for ciprofloxacin, 35.4% (*n*=17) for penicillin, 25% (*n*=12) for erythromycin and 10.4% (*n*=5) for tetracycline. The agreement analysis using Cohen’s kappa showed tetracycline exhibited the highest concordance with moderate agreement (κ=0.5, *P*<0.0003).

**Conclusions:**

Penicillin and erythromycin are used as first line agents for treatment and prophylaxis of diphtheria; however, our findings reveal high resistance rates among *C. diphtheriae* isolates in Karachi, Pakistan. This poses significant challenges for effective infection control, increasing the risk of persistent carriage and ongoing transmission. The observed multi drug resistance underscores the need for alternative therapeutic options and larger clinical studies correlating treatment outcomes with antimicrobial resistance to guide effective management.

